# Lymphocyte senescence in COPD is associated with loss of glucocorticoid receptor expression by pro-inflammatory/cytotoxic lymphocytes

**DOI:** 10.1186/s12931-014-0161-7

**Published:** 2015-01-09

**Authors:** Greg Hodge, Hubertus Jersmann, Hai B Tran, Mark Holmes, Paul N Reynolds, Sandra Hodge

**Affiliations:** Lung Research, Hanson Institute and Department of Thoracic Medicine, Royal Adelaide Hospital, Adelaide, South Australia; Department of Medicine, University of Adelaide, Adelaide, South Australia

**Keywords:** Lymphocyte senescence, COPD, Glucocorticoid receptor, CD28null T and NKT-like cells, IFNγ and TNFα

## Abstract

**Background:**

Glucocorticoid (GC) resistance is a major barrier in COPD treatment. We have shown increased expression of the drug efflux pump, Pgp1 in cytotoxic/pro-inflammatory lymphocytes in COPD. Loss of lymphocyte co-stimulatory molecule CD28 (lymphocyte senescence) was associated with a further increase in their pro-inflammatory/cytotoxic potential and resistance to GC. We hypothesized that lymphocyte senescence and increased Pgp1 are also associated with down-regulation of the GC receptor (GCR).

**Methods:**

Blood was collected from 10 COPD and 10 healthy aged-matched controls. Flow cytometry was applied to assess intracellular pro-inflammatory cytokines, CD28, Pgp1, GCR, steroid binding and relative cytoplasm/nuclear GCR by CD28+ and CD28null T, NKT-like cells. GCR localization was confirmed by fluorescent microscopy.

**Results:**

COPD was associated with increased numbers of CD28nullCD8+ T and NKT-like cells. Loss of CD28 was associated with an increased percentage of T and NKT-like cells producing IFNγ or TNFα and associated with a loss of GCR and Dex-Fluor staining but unchanged Pgp1. There was a significant loss of GCR in CD8 + CD28null compared with CD8 + CD28+ T and NKT-like cells from both COPD and controls (eg, mean ± SEM 8 ± 3% GCR + CD8 + CD28null T-cells vs 49 ± 5% GCR + CD8 + CD28+ T-cells in COPD). There was a significant negative correlation between GCR expression and IFNγ and TNFα production by T and NKT-like cells(eg, COPD: T-cell IFNγ R = −.615; ) and with FEV1 in COPD (R = −.777).

**Conclusions:**

COPD is associated with loss of GCR in senescent CD28null and NKT-like cells suggesting alternative treatment options to GC are required to inhibit these pro-inflammatory/cytotoxic cells.

## Background

Chronic obstructive pulmonary disease (COPD) is a leading cause of death world wide. Existing treatments are largely symptomatic and the use of anti-inflammatory corticosteroids has no proven disease modifying effect [1]. The mechanisms underlying this resistance, particularly in lymphocytes, are largely unknown [2] Our group has reported increased production of Th1 pro-inflammatory cytokines IFN-γ and TNF-α by CD8+ T cells in peripheral blood and lungs [3] and higher levels of the cytotoxic mediators granzyme b and perforin in peripheral blood in current and ex-smoker COPD patients compared to healthy smokers and never-smokers [4].

Our previous studies have focused on identifying the lymphocyte subset/s resistant to current therapeutics. We have shown that COPD is associated with increased CD8/CD28null cells in the peripheral blood of both current and ex-smoker COPD groups and these cells expressed more IFNγ, TNFα, granzyme B and perforin than CD8 + CD28+ cells [5]. CD8/CD28null oligoclonal T-cells have been reported in autoimmune diseases including granulomatous interstitial lung diseases [6] and in the aging immune system and its relationship to the development of COPD [7]. CD8/CD28null clones divide faster and live longer than CD8CD28+ T cells due to shorter cell division cycle, a higher resistance to apoptosis and a different response to regulatory cytokines [8].

Recently we also showed NKT-like and NK cells were increased in bronchoalveolar lavage (BAL) of COPD patients and this was associated with increased cytotoxicity by both cell types [9]. CD8 + CD28null NKT-like cells have been shown to be more pro-inflammatory and cytotoxic than CD8 + CD28+ NKT-like cells in lung transplant patients diagnosed with bronchiolitis obliterans syndrome [10], a further chronic inflammatory lung disease.

Importantly, the drug efflux pump, Pgp-1 was shown to be up-regulated in pro-inflammatory steroid resistant T and NKT-like cells in the peripheral blood of patients with COPD [11]. While elevated levels of Pgp-1 may contribute to a relative resistance to corticosteroids, it is likely that additional factors such as reduced levels of glucocorticoid receptors (GCR) in these pro-inflammatory lymphocytes may also play a role.

To investigate this hypothesis, we determined whether peripheral blood CD28null T cells (particularly CD8+) and NKT-like cells from COPD patients express reduced levels of GCR and/or increased Pgp-1 and whether loss of GCR (and/or increased Pgp-1) is associated with increased pro-inflammatory cytokines.

## Methods

### Patient and control groups

COPD volunteers were specifically recruited for the study and informed consent obtained. There was no exacerbation of COPD for 6 weeks prior. Subjects with other co-existing lung disease or malignancy or aged greater than 75y were excluded. Ethics approval was obtained from the Royal Adelaide Hospital and the experiments were conducted with the understanding and the written consent of each participant. COPD was diagnosed using the GOLD criteria with clinical correlation (mild COPD: FEV1/FVC < 70% but FEV1 ≥ 80% predicted; moderate COPD FEV1 50% ≤ 80% predicted, severe COPD FEV1 30% ≤ 50% predicted, very severe COPD FEV1 < 30%) [12]. Blood was collected from 10 patients with COPD (Table 1) all of whom were ex-smokers (at least one year) with an average of 37 pack years. No patients were receiving oral GCS.

**Table 1:** **Demographic details of the COPD and control group**

**Subjects**	**Controls**	**COPD**
No. of subjects	10	10
Age (years)	49 (±8)	58 (±16)*
FEV1, % pred	108.4 (±9)	60.1 (±20)
FEV1, % FVC	96 (±12)	58 (±15)*
Male/Female	8/6	6/4

Data showing mean ± SD.

*Abbreviations*: *FEV1* forced expiratory volume in 1 second, *FVC* forced vital capacity. *P < 0.05 compared to controls.

Blood was also obtained from 10 aged-matched non-smoking volunteers (Table 1) with normal lung function. These were healthy, recruited volunteers with no history of airways disease. All subjects underwent spirometry as part of their routine clinical assessment. Venous blood was collected into 10 U/mL preservative free sodium heparin (DBL, Sydney, Australia), and maintained at 4°C until processing. All patients were submitted to the same protocol and analysis performed retrospectively.

### Leucocyte stimulation

Leucocyte stimulation was required for intracellular cytokine and Pgp1 expression [11,13]. Consistent with previous reports [14,15] preliminary experiments showed GCR expression was detected in less than 2% of unstimulated T cells but GCR expression was significantly upregulated in T and NKT-like cells following the same stimulation as for intracellular cytokine and Pgp1 detection. One mL aliquots of blood diluted 1:2 with RPMI 1640 medium (Gibco, New York, USA) supplemented with 125 U/mL penicillin and 125 U/mL streptomycin) were placed in a 10 mL sterile conical PVC tubes (Johns Professional Products, Sydney, Australia). Phorbol myristate (25 ng/mL) (Sigma, Sydney, Australia) and ionomycin (1 μg/mL) (Sigma) was added. Brefeldin A (10 μg/mL) (Sigma) was added as a “Golgi block” and the tubes re-incubated in a humidified 5% CO_2_/95% air atmosphere at 37°C for 16 h. Preliminary studies showed that addition of Brefeldin A was shown not to affect Pgp1 or GCR expression in T and NKT-like cells (not shown).

### Pgp1 and GCR expression by CD28+ and CD28null T, NKT-like cells

Following stimulation as described above, 350 μL aliquots of cells were treated with 2 mL FACSLyse (BD Bioscience, Sydney, Australia) for 10 min. Cells were centrifuged, supernatant discarded and 500 mL FACSPerm (BD) added for 10 min. Two mL 0 · 5% bovine serum albumin (BSA) (Sigma) in IsoFlow (Beckman Coulter, Sydney, Australia) was then added and the tubes centrifuged at 300 *g* for 5 min. After decanting supernatant, Fc receptors were blocked with 10 mL human immunoglobulin (Intragam, CSL, Melbourne, Australia) for 10 min at room temperature. Five μL of mouse anti-human GCR (clone 5E4, Serotec, Sydney, Australia; raised against a conserved sequence of the regulatory part of the receptor- amino acids 150–176) as previously reported [16] was added to cells for 15 min, and following washing (as above), 5 μL rat anti-mouse IgG1 V450 (BD) was added for 15 min. Following washing, 5 μL of appropriately diluted CD3 perCP.Cy5.5 (BD), Pgp1 PE (BD), CD28 PECY7 (BD), CD56 APC (Beckman Coulter), CD8 APCH7 (BD) and CD45 V500 (BD) were added for 15 min in the dark at room temperature. Cells were washed and events acquired and analyzed as previously reported [11,13].

### Pgp1, GCR, IFNγ and TNFα expression by CD28+ and CD28null T, NKT-like cells

To determine possible association of pro-inflammatory cytokines and Pgp1 and GCR expression by CD28+ and CD28null T and NKT-like cells, whole blood was stimulated as described above. Following stimulation and processing, cells were labeled with anti-GCR as described above, then 5 μL of appropriately diluted IFNγ FITC (BD), TNFα FITC (BD), Pgp1 PE (BD), CD3 perCP.Cy5.5 (BD), CD28 PECY7 (BD), CD56 APC (Beckman Coulter), CD8 APCH7 (BD) and CD45 V500 (BD) were added for 15 min in the dark at room temperature. Cells were washed and events acquired and analyzed as described [5,11,13].

### Correlation of GCR with steroid binding capacity in CD28+ and CD28null T and NKT-like cells

To correlate steroid binding with GCR expression of CD28+ and CD28 null T and NKT-like subsets, 350 μL aliquots of cells following stimulation as described above were added to 10^−5^ M Dexamethasone (Hospira, Melbourne, Victoria, Australia) for 30 min in a humidified 5% CO_2_/95% air atmosphere at 37°C followed by 5 μL Dexamethasone Fluorescein (Molecular Probes, Life Technologies, Sydney, Australia) for a further 30 min in a humidified 5% CO_2_/95% air atmosphere at 37°C. Cells were then washed, stained with monoclonal antibodies and analysed as described above.

### Cytoplasm/nuclear GCR expression by CD28+ and CD28null T, NKT-like cells

To determine the location of GCR expression in CD28+ and CD28null T and NKT-like cells differential staining of whole blood following stimulation (as described above) using reagents to sequentially permeabilise the cytoplasm and nucleus as previously described [17]. Briefly, following stimulation, 350 μL aliquots of stimulated whole blood were treated with FACSLyse as described above and following centrifugation cell cytoplasmic membrane was permeabilised with 0.1% saponin for 10 mins. Following centrifugation, cells were resuspended in 100 μL 0.1% saponin then labeled with anti-GCR as described above. Following washing in 0.1% saponin, cells were stained with rat anti-mouse IgG1 V450 (BD) for 10 min. After washing in 0.1% saponin, the cells were permeabilised with 500 μL 0.1% Triton in PBS for 10 min. Cells were then incubated with anti-GCR as described above, followed by rat anti-mouse IgG1 PE for 10 min. After washing in 2 mls 0.5% BSA in FACSFlow, cells were stained with 5 μL of appropriately diluted CD3 perCP.Cy5.5 (BD), CD28 PECY7 (BD), CD56 APC (Beckman Coulter), CD8 APCH7 (BD) and CD45 V500 (BD) for 10 min. After washing, data was acquired as described above.

### GCR expression in CD28+ and CD28null T cells by Fluorescent Microscopy

PBMC were isolated from blood of a cohort of 3 control and 3 COPD patients by standard density gradient centrifugation and cells re-suspended at 1 × 10^7^ mL in RPMI 1640 medium. Following stimulation as described above, 25 μL of appropriately diluted CD3 perCP.CY5.5 (BD), CD28 PE.CY7 (BD), CD56 APC (Beckman Coulter), CD8 APC.CY7 (BD) and CD45 V500 (BD) monoclonal antibodies were added for 15 min in the dark at room temperature. Cells were washed and resuspended in 1 mL RPMI and CD28+ and CD28null, CD8+ and CD8- T cells were immediately sorted on a FACSAria flow cytometer (BD).

1 × 10^3^ sorted CD28+ and CD28 null T cells were added to a Cytospin 4 cytocentrifuge (ThermoFisher Scientific, Scorseby, Victoria, Australia) and centrifuged for 500 *g* for 5 min. Slides were air dried for 10 min and cells fixed with 2.5% formalin in PBS for 10 min. Cytospins were treated with 1% sodium dodecyl sulphate (SDS, Sigma Aldrich, Castle Hill, NSW, Australia) in PBS for 5 min, followed by 1 h incubation with a serum-free protein blocker (Dako A/S, Glostrup, Denmark), overnight incubation with 1/25 diluted GCR monoclonal antibody (Serotec, Abacus ALS, Brisbane, Australia), then 1 hour with AF549-conjugated goat polyclonal antibody to mouse IgG1 (Invitrogen, Thornton, NSW, Australia), and counterstained with DAPI (Sigma-Aldrich). Cells were washed between incubation with 0.01 M Tris-buffered saline pH 7.5, containing 0.05% Tween-20. Immunofluorescence was detected and imaged with a Zeiss fluorescence microscope equipped with HBO 100 illuminating system, AxioCam MRn digital camera and AxioVision 4.8.1 software (Carl Zeiss GmbH, Goettingen, Germany).

### Statistical analysis

Statistical analysis was performed using the Mann–Whitney test. For T-cell subsets CD28null/CD8+/CD3+/CD56-/CD45+/TNFα+/IFNγ+), a sample size of n = 10 allowed a power of 98–99.5% for analysis. Variance was estimated from our previous studies [3-5,9-11]. Correlations were performed using Spearman Rho correlation tests. SPSS software was applied and differences between groups of p < 0.05 considered significant.

## Results

### Increased CD28null CD8+ T and NKT-like cells in COPD patients

There was a significant increase in CD28null CD8+ T in patients with COPD compared with healthy controls but no change in CD28null CD8- T cells (CD28nullCD8 + T: 55 ± 7.8 (35 ± 6.3); CD28nullCD8-T: 7.5 ± 2.9 (6.3 ± 2.6) for COPD patients (controls) (mean ± sd)) consistent with our previous findings for CD28null T cells [5]. There was a significant increase in CD28null CD8+ NKT-like cells in patients with COPD compared with healthy controls but no change in CD28null CD8- NKT-like cells (CD28nullCD8 + NKT-like: 38 ± 6.4 (23 ± 4.3); CD28nullCD8-T: 8.3 ± 3.1 (7.4 ± 2.8) for COPD patients (controls)).

### Pgp1 expression by CD28+ and CD28null T, NKT-like cells

**Figure 1:**
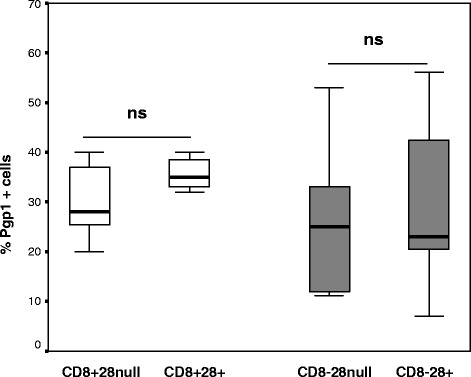
**The percentage of CD28+ and CD28null CD8+ (clear bars) and CD8- T cells expressing Pgp1 in patients with COPD.** Data presented as box plots. There was no significant change in the percentage of Pgp1+ CD8 + CD28null and CD8 + CD28+ or CD8-CD28null and CD8-CD28+ T cells in patients with COPD (p > 0.05 for all).

There were no significant differences in Pgp1 expression by stimulated CD28+ or CD28 null CD8- or CD8+ CD3+ T or NKT-like cells from either COPD patients or controls (data for COPD T cells shown in Figure 1) ( Data for controls and NKT-like cells not shown).

### GCR expression by CD28+ and CD28null T and NKT-like cells

**Figure 2:**
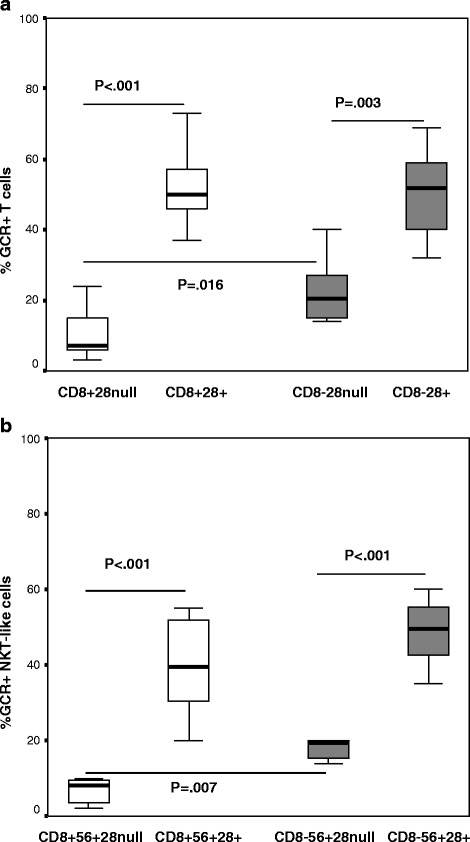
**The percentage of CD28+ and CD28null CD8+ (clear bars) and CD8- NKT-like cells expressing GCR in patients with COPD.** **a**. The percentage of CD28+ and CD28null CD8+ (clear bars) and CD8- T cells expressing GCR in patients with COPD. Data presented as box plots. There was a significant decrease in the percentage of CD28null/CD8- and CD28nullCD8+ T cells expressing GCR compared with CD28 + CD8- and CD28 + CD8+ T cells. There was a significant decrease in the percentage of CD8 + CD28null T cells expressing GCR compared with CD8-CD28null T cells. **b**. The percentage of CD28+ and CD28null CD8+ (clear bars) and CD8- NKT-like cells expressing GCR in patients with COPD. Data presented as box plots. There was a significant decrease in the percentage of CD28null/CD8- T and CD28nullCD8+ NKT-like cells expressing GCR compared with CD28 + CD8- T and CD28 + CD8+ NKT-like cells. There was a significant decrease in the percentage of CD8 + CD28null NKT-like cells expressing GCR compared with CD8-CD28null T cells.

There was a significant decrease in the percentage of CD28null/CD8- and CD28nullCD8+ T cells and NKT-like cells expressing GCR in both COPD groups and controls compared with their CD28+ T and NKT-like cells (Data for T cell and NKT-like cell subsets from COPD group shown in Figure 2a and b respectively) (data for controls not shown). There was a significant decrease in the percentage of CD8 + CD28null T cells expressing GCR compared with CD8-CD28null T cells (Figure 2a) and NKT-like cells (Figure 2b) from COPD patients and controls (Data for controls not shown).

### Correlation between GCR and IFNγ and TNFα production in T and NKT-like cells

**Figure 3:**
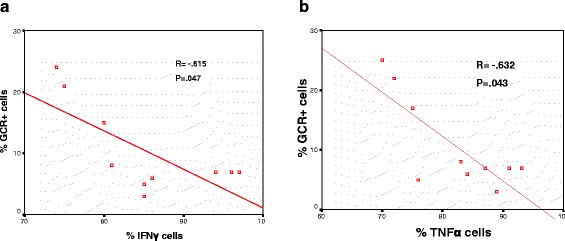
**There was a significant negative correlation between GCR expression and the percentage of CD8 + CD28null T cells producing IFNγ** **(a) and TNFα** **(b) from COPD patients.**

There was a significant correlation between GCR expression and the percentage of CD8 + CD28null T cells and NKT-like cells producing IFNγ (Figure 3a) and TNFα (Figure 3b) from both COPD groups and controls (data for controls and NKT-like cells not shown).

### Correlation between GCR and steroid binding in T and NKT-like cells

**Figure 4:**
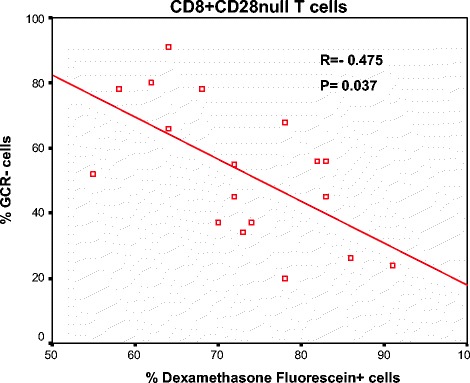
**There was a significant negative correlation between the percentage of GCR negative CD8 + CD28null T cells and the percentage of Dexamethasone Fluorescein positive cells from 10 COPD patients and 8 control subjects.**

Low GCR expression correlated with low Dex-Fluor binding for both COPD patients and control subjects (Figure 4) and NKT-like cells (data not shown).

### GCR expression of CD28+ and CD28null T cells by Fluorescent Microscopy

**Figure 5:**
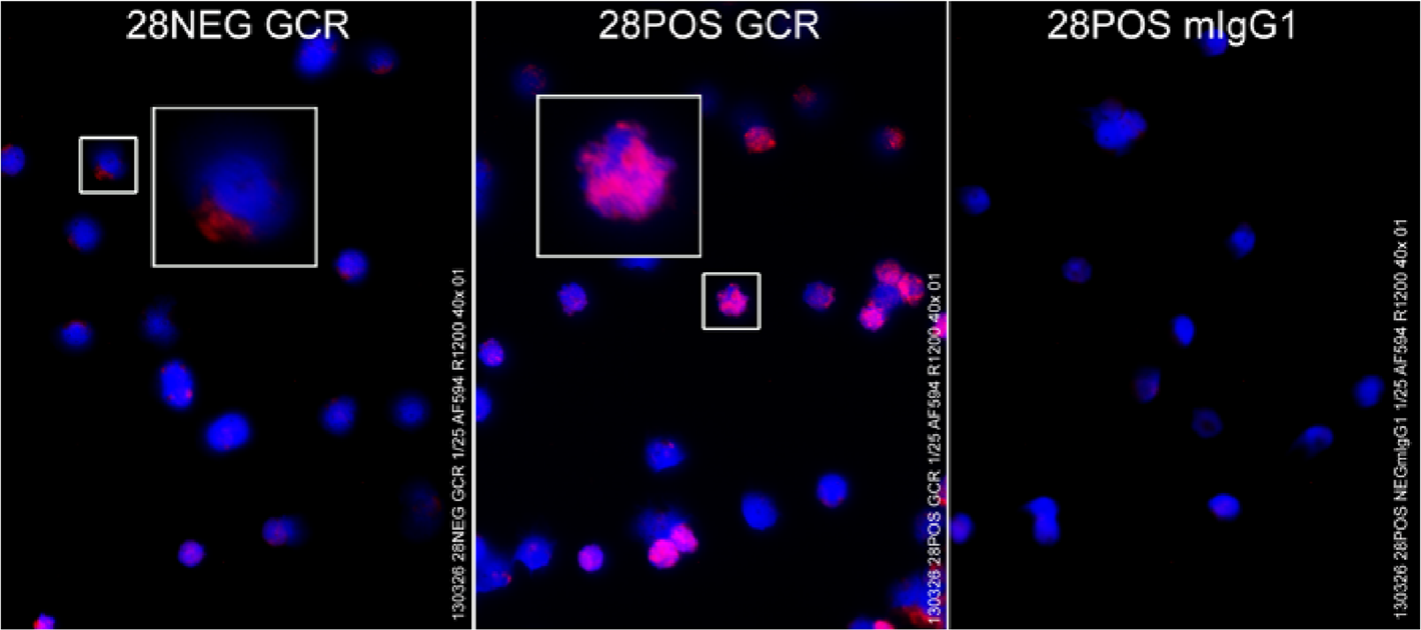
**Representative immunofluorescence microphotos of GCR staining in sorted CD28null (left) and CD28+ T cells (middle).** Magenta is merged color of red (GCR) and blue (DAPI). Mean fluorescence intensity measured by ImageJ software was 160.5 ± 9.3 in CD28null and 336.2 ± 25.0 in CD28+ cells (p < 0.001). Experiments were repeated 2 times, showing similar results.

Sorted CD28+ and CD28null T cells were stained for GCR expression. There was significant positive staining with GCR in CD28+ T cells compared with CD28 null T cells using fluorescence microscopy (Figure 5). GCR staining was mainly located in the CD28+ T cell nucleus (Figure 5).

### GCR expression in the cytoplasm and nucleus of CD28+ T cells compared with CD28 null T cells

**Figure 6:**
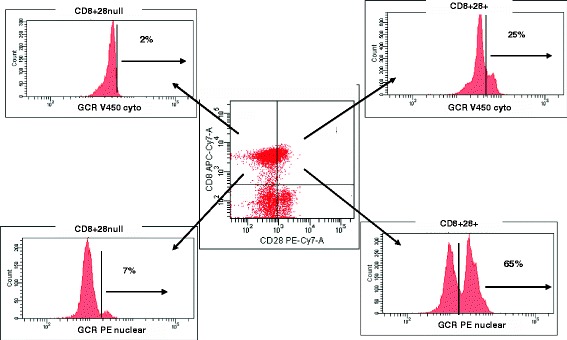
**Representative flow cytometry plots showing expression of GCR in CD8 + CD28null and CD8 + CD28+ T cells in the cytoplasm and nucleus following stimulation.** There was a significant increase in GCR expression in the cytoplasm and nucleus of CD8 + CD28+ cells compared with CD8 + CD28null cells (p < 0.05 for all, from 5 experiments).

To confirm nuclear staining of GCR in CD28+ T cells, differential expression of GCR in the cytoplasm and nucleus of CD28+ and CD28null T and NKT-like cells was performed. There was a significant increase in GCR expression in the cytoplasm and nucleus of CD8 + CD28+ cells compared with CD8 + CD28null cells although the proportion of GCR expression in the cytoplasm/nucleus of both subsets was similar. Representative flow cytometry plots showing expression of GCR in CD8 + CD28null and CD8 + CD28+ T cells in the cytoplasm and nucleus following stimulation are shown in Figure 6.

### Correlation between GCR and FEV1

**Figure 7:**
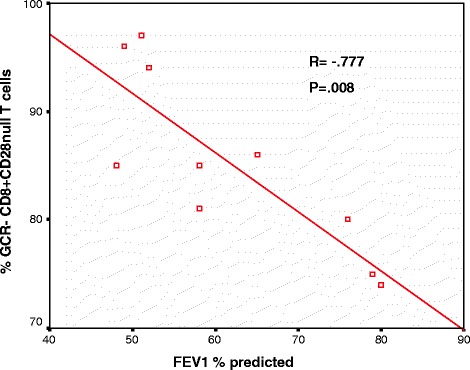
**There was a significant negative correlation between FEV1 % predicted and the percentage of GCR negative CD8 + CD28null T cells in COPD patients.**

There was a significant negative correlation between FEV1 of COPD patients and the percentage of GCR negative CD8 + CD28null T cells (Figure 7) and NKT-like cells (data not shown).

## Discussion

Lymphocyte senescence and GC resistance have been described in several other inflammatory conditions such as cardiovascular disease [18], autoimmune disease [19], arthritis [20], IBD [21] associated with aging [22] and aging with associated inflammation in COPD [23]. This is the first study to show that lymphocyte senescence in COPD is associated with a loss of GCR from both T and NKT-like cells. Importantly, the loss of GCR by increased numbers of senescent CD8 + CD28null T cells and NKT-like cells was shown to correlate with COPD disease severity.

We have previously shown an increase in pro-inflammatory CD8+ T cells in peripheral blood and lungs [3] and an increase in cytotoxic NKT-like and NK cells in the airways in COPD patients compared to healthy and never-smokers [9]. Consistent with this we identified increased CD8/CD28null cells in both current and ex-smoker COPD groups [5]. Senescent CD28null T and NKT-like cells have been shown to be more pro-inflammatory and cytotoxic than their CD28 positive counterparts [5,9], and exhibit a relative resistance to corticosteroids [10]. The mechanism for this resistance was at least partially explained by our findings of an increase in the drug efflux pump Pgp1 in pro-inflammatory peripheral blood T and NKT-like cells [11]. However, we did not differentiate Pgp1 expression on CD28+ and CD28null T and NKT-like cells in this previous study. We also hypothesized that steroid resistant senescent lymphocytes may have decreased levels of GCR. There have been reports of reduced GCR number in PBMC in patients with steroid resistant asthma [24] and importantly, reduced GCRα protein expression in the peripheral lung of patients with COPD and smokers with normal lung function [25]. However, there have been no reports of decreased GCR expression in the peripheral blood of patients with COPD, or in the various lymphocyte subsets. Our current study shows there is a significant loss of GCR expression in CD28null T and NKT-like subsets, with the greatest loss demonstrated in CD8 + CD28null cells, the senescent and most pro-inflammatory subset [5]. Importantly, these GCR deficient lymphocytes were shown to be present in the systemic circulation of COPD patients. Barnes et al. proposed a spillover of cells from the lungs into the systemic circulation [2], which suggest these GCR deficient cells may have originated in the lung. There have been conflicting reports that either support or dispute this concept. One report showed no difference in GCR expression in lung follicular CD8 cells between COPD patients and smokers [26] using immunofluorescence techniques. This study did not however differentiate between GCR expression in CD28+ and CD28null subsets of CD8 T cells. Another study of patients with interstitial lung disease showed decreased expression of GCR mRNA in lung tissue from steroid resistant compared with steroid sensitive patients [27].

We then investigated the intracellular localization of GCR in the various lymphocyte subsets and showed reduced cytoplasmic and nuclear GCR expression in CD28null compared with CD28+ T and NKT-like cells. Following stimulation there was a similar proportion of GCR in the cytoplasm and nucleus of both CD28null and CD28+ CD8+ T cells from both COPD patients and control subjects, suggesting there is no defect in nuclear translocation of the GCR between these subsets. Interestingly, our present study showed that a loss of GCR expression by CD28null T and NKT-like cells was also observed in healthy control subjects although at decreased numbers compared with patients with COPD ie., GCR expression was the same in CD28null T and NKT-like cells from both subject groups. One could speculate that these cells may be the precursors to potential inflammatory diseases. Our findings suggest that the relative GC resistance of the CD28null inflammatory lymphocytes need to be considered with any therapeutic approaches, and alternative therapies to GC may be required to avoid susceptibility to inflammatory diseases. To our knowledge, there have been no treatments to date that have increased lymphocyte GCR expression, although there has been a report of successful pitavastatin treatment of acute lung injury in septic mice with associated increase in GCR in alveolar macrophages [28] and in studies using selective GCR agonists in the treatment of inflammatory bowel disease [29]. Investigation of the possible effects of these therapeutics on GCR expression in CD28null CD8+ T cells would be worthwhile. We have previously shown CD137 is increased in CD28null T and NKT-like cells in COPD [30]. In this previous study there was a significant decrease in the percentage of CD28null T and NKT-like cells producing IFNγ and TNFα in the presence of anti-CD137 blocking antibody compared with CD28+ T and NKT-like counterparts suggesting these pro-inflammatory cells may be targeted therapeutically. In conclusion, lymphocyte senescence in COPD is associated with loss of GCR in CD28null T and NKT-like cells. This loss is related to disease severity in COPD, thus therapies aimed at increasing GCR expression in pro-inflammatory senescent lymphocytes are warranted.
